# Genomic-Encoded Mitovirus RdRp Is Required for Embryo Development and Maintaining Mitochondrial Dynamics in *Arabidopsis*

**DOI:** 10.3390/ijms26189035

**Published:** 2025-09-17

**Authors:** Yadi Gong, Rongqin Chen, Chen Yang, Yingcui Lu, Zhenjie Fu, Ye Feng, Xiaomeng Li, Ling Li, Xiaoyun Li

**Affiliations:** Guangdong Provincial Key Laboratory of Biotechnology for Plant Development, School of Life Science, South China Normal University, Guangzhou 510631, China

**Keywords:** RNA-dependent RNA polymerase (RdRp), mitovirus, mRdRp, *Arabidopsis*, coevolution

## Abstract

Mitoviral-derived sequences are frequently detected in plant genomes, encoding an RNA-dependent RNA polymerase (RdRp). These sequences share many similarities with mitoviruses that are known to commonly infect plant mitochondria. However, the functional characterization of nuclear-encoded mitoviral-RdRp remains unclear. This study elucidates the critical role of mRdRp (AT2G07749) in maintaining mitochondrial homeostasis and embryo viability, highlighting the dual role of viral-derived genes in plant development and stress response. Phylogenetic analysis reveals that mRdRp shares 96.8% identity with the mitoviral RdRp encoded by mitochondrial-genomes, suggesting that this nuclear *mRdRp* gene originated from horizontal transfer events following ancestral plant-mitovirus infections. To dissect mRdRp function, we generated a *mRdRp* knockout mutant via CRISPR-Cas9 or knockdown mutant by RNA interference (RNAi). These *mRdRp* mutants exhibited severe developmental defects, including dwarfism, embryo lethality, and sterility. Phenotypic assays further showed that *mRdRp* mutants displayed heightened susceptibility to ABA and rotenone, indicating impaired adaptive capacity to both hormonal and metabolic stress. Loss of mRdRp led to fragmented mitochondrial networks and a significant reduction in mitochondrial abundance in both leaf protoplasts and root meristematic cells. Additionally, mitochondrial-derived small RNA (sRNA) aberrantly accumulated in *mRdRp* mutants, which potentially disrupts endogenous RNA-silencing pathways that rely on sRNA-mediated gene regulation. Collectively, these results provide mechanistic insights into the function integration of a virus-derived gene into plant cellular networks, advancing our understanding of host–virus coevolution and the role of horizontally transferred viral genes in shaping plant physiology.

## 1. Introduction

Mitochondria are dynamic and highly plastic organelles that can quickly change their shape and function in response to cellular demands [1,2]. They play a significant role in various cellular life processes, including energy metabolism, ion homeostasis, lipid droplet formation, redox signaling, and cell death, among others. As organelles of endosymbiosis origin, mitochondria have undergone significant gene loss or transfer to the nuclear genome throughout evolution [3,4]. Over a thousand nuclear genes in humans are derived from mitochondrial ancestors [5,6]. Fragments of mitochondrial DNA escape (e.g., through membrane rupture or extracellular vesicles) and integrate into nuclear genomes via non-homologous recombination and form pseudogenes [7,8]. Intriguingly, some transferred genes acquire nuclear promoters and splicing signals, allowing for functional expression [4]. Thus, their uniform nuclear-coordinated expression reduces mitochondrial genome complexity and enhances nuclear control over cellular energetics. Flowering plants also acquire mitochondrial genes frequently via horizontal transfer; this process optimizes metabolic regulation, enhances nutrition acquisition and stress-resistance, or improves productivity [4,9,10]. For example, the mitochondrial genome encodes only a few subunits of cytochrome c oxidase and ATP synthase, with the majority being encoded by the nucleus [11].

Moreover, plant mitochondrial genomes are notably larger and more unstable in angiosperms [12,13]. These genomes exhibit extensive genetic polymorphism and a reduced number of mitochondrial protein-coding genes, a consequence of frequent losses of ribosomal protein genes and succinate dehydrogenase genes [14]. Numerous research findings indicate that a large number of nuclear-encoded mitochondrial proteins are involved in plant development and their mutants exhibit significant growth impairment. These include mutants related to mitochondrial synthesis (e.g., *mrpl1* [15], *cls* [16], *mterf15* [17], *mterf18* [18], and *mterf22* [19], mitochondrial transport (*ndt2* [20], *tim17-1* [21], *atvdac2* [22], *ropgef2* [23]), mitochondrial RNA editing (*abo6* [24], *ahg11* [25], *asd* [26], *abo5* [27], *abo8* [28], *pgn* [29], *ppr40* [30], *slg1* [31], *slg2* [32], *slo3* [33]), mitochondrial metabolism (*mmdh1* [34], *mmdh2* [35], *icl* [36], *fum1* [37]), mitochondrial distribution and mitophagy (friendly [38,39,40]), among others. Among these genes, most mitochondrial-related genes are required for embryo development. In rice, many nuclear-encoded mitochondrial proteins have been associated with cytoplasmic male sterility (CMS), such as COXII [41], PPR [42]. These findings further suggest that mitochondria are essential for the development of gametophytes or embryos during the reproductive process.

Mitoviruses are the simplest viruses with genomes encoding only a single RNA-dependent RNA polymerase (RdRp), and are widespread across fungi, plants, and invertebrates [43,44,45,46,47]. Mitoviruses appear to be horizontally transferred from plant pathogenic fungi [44]. Additionally, numerous mitovirus-derived sequences are present within the plant genome [44]. In *Arabidopsis*, four mitovirus-like RdRp homologs have been identified: three are mitochondrial-encoded members (ATMG01410, ATMG01110, and ATMG00490), and one is nuclear-encoded (AT2G07749). AT2G07749 shares 96.81% sequence identity with ATMG01110, suggesting that AT2G07749 may have been horizontally transferred from mitochondria. AT2G07749 encodes a mitochondria-localized protein of 244 amino acids (AA), designated as mRdRp. This protein is expressed in various tissues, including seeds, root tips, leaf veins, and senescent leaf stalks (as indicated by the TAIR database). Whether the ancestral mRdRp (putatively derived from mitoviruses) has functions related to plant health remains unknown. Here, we report the functional characterization of mRdRp in maintaining mitochondrial dynamics during embryo development in *Arabidopsis.*

## 2. Results

### 2.1. Phylogenetic Analysis of mRdRp

**Figure 1:**
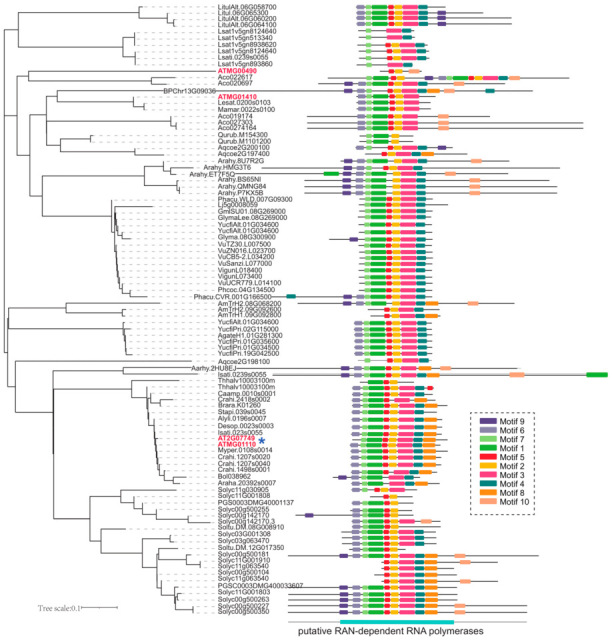
Phylogenetic relationships and conserved motifs of mRdRp. The construction of a phylogenetic tree using the neighbor-joining method, with Bootstrap values set to 1000 and other parameters maintained at their default settings. The red font represent these gene from *aribidopsis*. * Asterisks indicated the mRDRP (AT2G07749) and its mitochondrial-encoded homologs (ATMG01110).

To investigate the diversification among mitoviruses and their horizontally transferred genes, a phylogenetic tree was constructed based on the amino acid sequences of 95 genes from 15 species by using the MEGA-11 software (Figure 1). Total night conserved motifs were predicted for these proteins using the MAST tool (https://meme-suite.org). The mitovirus-like genes are primarily categorized into two major groups: those encoded by the mitochondria and those encoded by the nucleus. They possess different lengths or motif numbers, suggesting that they may have undergone different evolutionary pressures. In various species, the nuclear-encoded copies are more homologous and primarily contain eight motifs for a complete, putative RNA-dependent RNA polymerase [48], such as AT2G07749 (mRdRp) and other mRdRp from various species’ nuclear-encoded copies (Figure 1). The mRdRp exhibited a similar identity (96.81%) to the mitochondrial copy ATMG01110, suggesting functional conservation for viral replication or the evolution of a new function.

### 2.2. Loss Function of mRdRp Severely Impairs Plant Growth

**Figure 2:**
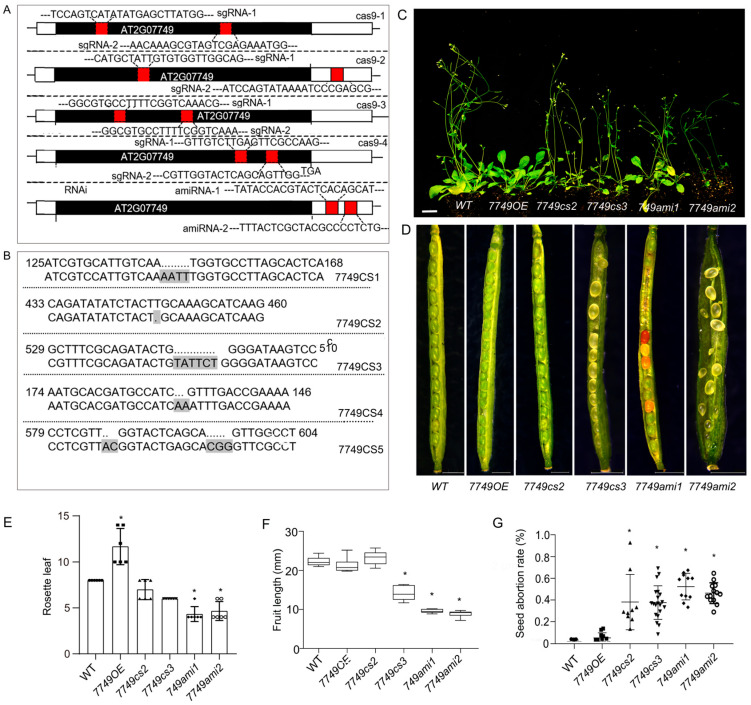
Phenotype of *mRdRp* mutants. (**A**), Schematic of *mRdRp* knockout or knockdown strategies. Four CRISPR/Cas9 constructs (*cas9-1* to *cas9-4*) with distinct guide RNAs (sgRNAs) and two artificial microRNA constructs (*amiRNA1*, *amiRNA2*) were designed to target non-redundant regions of the *mRdRp* genes. (**B**), Validation of *mRdRp* mutants by PCR-sequencing. Five independent *mRdRp* mutants (*7749cs1*–*5*) were identified, with each line carrying a unique frameshift or missense mutation in *mRdRp* coding sequence. The gray area indicates that the bases have been deleted in these mutants. (**C**), Growth phenotype of *mRdRp* mutants at the vegetative stage. Compared to the wild type (WT), four-week-old *mRdRp* mutant lines exhibit significant dwarfism and infertility. Scale bar = 1 cm. (**D**), Fertility phenotype of the *mRdRp* mutants. WT produced fully developed siliques with plump seeds, whereas mutant lines (e.g., *7749 cs3*, *7749 ami1*, and *7749 ami2*) showed shortened siliques and visible seed abortion. Scale bar = 1 cm. (**E**–**G**), The phenotype of the *mRdRp* mutant, including rosette leaf amount, mature fruit length, and seed abortion rate. Bars are the means (±SD) of three biological replicates (n = 10). Statistical significance was determined by one-way analysis of variance (ANOVA) with Dunnett’s post hoc test; * *p* < 0.01, indicates significant differences between mutants and WT.

Five *mRdRp* knockout mutants were created at various sites using CRISPR-Cas9 (Figure 2A,B). Compared to the wild type, these mutants displayed slower growth during the seedling stage, which resulted in a dwarf phenotype and a reduced seed yield (Figure 2C,D). Among them, the phenotype of *7749cs2* line was slightly milder, while the other knockout mutants were similar to the *7749cs3* line, exhibiting a relatively severe case of dwarfism and embryo death (Figure 2C,D). Furthermore, the expression of *mRdRp* was diminished through RNA interference, leading to more severe growth defects than those seen in knockout mutants, such as stunted plants and infertility (Figure 2C,D). At least ten RNAi lines were obtained; however, most of them could not produce normally germinated seeds. The *mRdRp* mutants exhibited significant reductions in mature siliques length, number of seeds per pod (Figure 2E–G). However, overexpression of mRdRp did not result in any obvious phenotype growth, except for some late blooming (Figure 2E–G). Notably, compared to the knockout lines, two RNAi mutant lines (*7749ami1* and *7749ami2*) were able to harvest some normal germinating seeds and exhibited a relatively stable phenotype. Consequently, they were selected for further stress-response experiments. Take together, these results indicate that the nuclear-encoded mRdRp plays an important role in *Arabidopsis* growth.

### 2.3. Loss Function of mRdRp Severely Impairs Plant Stress Resistance

**Figure 3:**
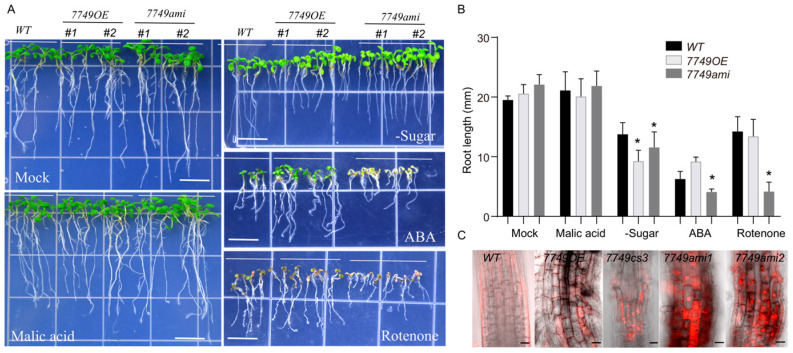
The stress resistance of *mRdRp* mutant. (**A**), Photographs of seedlings grown for seven days after stratification on agar plates containing various substances, including 10 μM malic acid, 50 μM ABA, and 10 μM rotenone. (**B**), The statistical analysis of root length in (**A**), bars indicate standard deviation, n = 10, * indicate a significant difference between mRdRp and mRdRp plants compared with WT plants (*p* < 0.01). (**C**), Photographs of roots under confocal microscopy. PI represent both the cell wall and apoptosis, and when it stains the nucleus, the cell undergoes apoptosis. Scale bar = 20 μm.

As a plant hormone, abscisic acid (ABA) mediates plant responses to various stresses and is frequently utilized to indicate plant stress resistance. After exposure to ABA, the *mRdRp* mutant displayed severely stunted growth (Figure 3A,B), indicating that stress resistance should be impaired in these plants. Generally, the *mRdRp* mutant exhibits rapid growth under normal conditions but does not reach a significantly different result on the agar plate (Figure 3A). It may be that the presence of mild apoptosis in *mRdRp* mutants promotes root growth (Figure 3C). The growth of the *mRdRp* mutant is stunted in the absence of sugar or when exposed to Rotenone (Figure 3A,B). Note that the overexpression of RdRp in plants also exhibited sensitivity to sugar deficiency (Figure 3A,B). However, no significant difference in growth is observed when exposed to an appropriate level of malate, an intermediate product of the TCA cycle. This indicates that oxidative phosphorylation in the mitochondria is affected in *mRdRp* mutants.

### 2.4. Loss Function of mRdRp Severely Impairs Mitochodnria Dynamics

**Figure 4:**
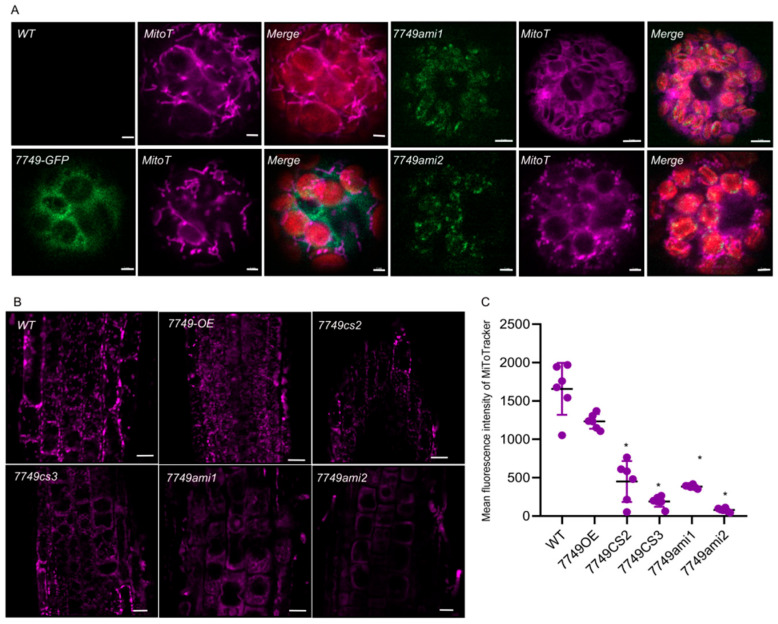
mRdRp affects the dynamics of mitochondria. (**A**), Subcellar localization of mRdRp and its impact on mitochondrial morphology in protoplasts. Left panel, protoplasts transiently expressing mRdRp-GFP (green) and the mitochondria were marked using MitoTracker (1 μM, purple). Right panel, protoplast expressing amiR-mRdRp (to knockdown mRdRp) exhibit disrupted mitochondrial morphology (compared to mRdRp-GFP-expressing or WT protoplasts). Scale bar = 2 μM. (**B**), Mitochondrial morphology in root tip cells of WT and *mRdRp* mutants. Confocal images of MitoTracker-stained root cells (meristematic zone, 5-day-old seedlings). WT roots show long and big mitochondrial, whereas mutant roots display small, scattered mitochondrial. Scale bar = 20 μm. (**C**), Quantitative analysis of mitochondria in WT and mutant roots. Fluorescence intensity is 50–60% lower in *mRdRp* mutant roots cells than in WT (*p* < 0.01). Bars represent the meant (±SD) of three biological replicates (n = 6 root tips per line). Statistical significance was determined by one-way ANOVA with Dunnett’s post hoc test. * *p* < 0.01, indicates significant differences between mutants and WT. Scale bar = 20 μm.

The *mRdRp-GFP* and *amiR-mRdRp* were transformed into protoplasts. The expression of mRdRp-GFP was clearly localized in the cytoplasm (Figure 4A). No significant alterations in the mitochondria were observed in the mRdRp-GFP-transformed protoplasts compared to the control groups. In contrast, the mitochondria in the *amiR–mRdRp*-transformed protoplasts were dramatically dispersed and reduced (Figure 4A). Furthermore, mitochondria were observed in the roots from various lines. Consistent with the protoplast results, overexpressed *amiR–mRdRp* commonly reduced mitochondrial quality (Figure 4B,C). These results indicate that mRdRp plays an important role in maintaining mitochondrial dynamics.

### 2.5. Loss Function of mRdRp Elevates Small RNA Expression in Mitochondria

**Figure 5:**
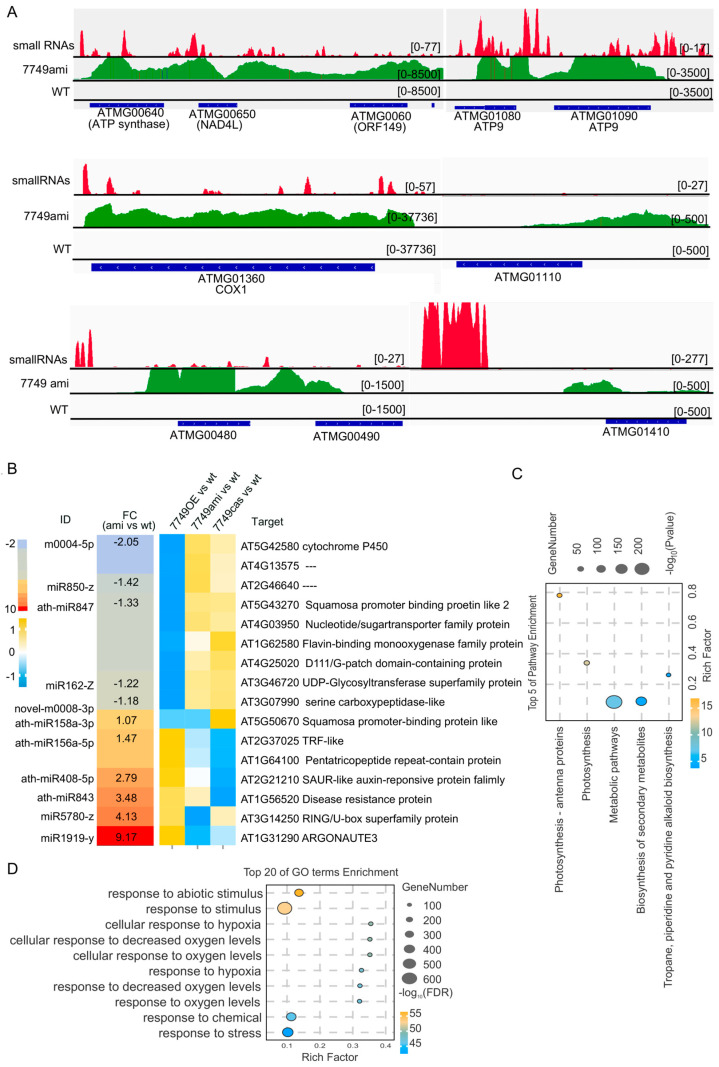
Numerous small RNA were produced in a *mRdRp* mutant. (**A**), Small RNA and RNA sequencing were conducted on the *mRdRp* mutant. (**B**). The fold changes in miRNA and its targeted genes’ expression in the *mRdRp* mutant. (**C**,**D**). KEGG and GO analysis of the 1526 genes with decreased expression in the *mRdRp* mutant.

To dissect the role of mRdRp in sRNA regulatory networks, we performed genome-wide sRNA sequencing on mitochondria-enriched fractions and total RNA from *mRdRp* mutants. This analysis uncovered a striking, previously unreported phenotype, a massive accumulation of mitochondrial-derived sRNA (mt-sRNAs) in *mRdRp* mutants. These enriched mt-sRNAs were predominantly 21–24 nt in length and main derived from two primary regions: (1) protein-coding genes, primary subunits of the mitochondrial ATP synthase and COXI; and (2) intergenic regions (Figure 5A). However, the function of these small RNAs remains unknown. Beyond mt-sRNAs, we observed significant dysregulation of nuclear-encoded microRNA (miRNAs) in *mRdRp* mutants (Figure 5B). Among these, *miR1919-Y*, a miRNA previously linked to RNA silencing of the *argonaute (AGO)* gene, was significantly highly upregulated (9.17-fold vs. WT). Consistent with this, transcriptome analysis showed that the expression of AGO3 was reduced by 62% in *mRdRp* mutants. Since AGO3 is a core component of the RNA-induced silencing complex (RISC) that mediates sRNA-dependent gene silencing, its downregulation likely amplifies sRNA-mediated regulatory defects in *mRdRp* mutants. To link sRNA dysregulation to the developmental and stress related phenotypes of *mRdRp* mutants, we integrated sRNA seq data with transcriptome profiling. A total of 1526 nuclear genes were significantly downregulated in *mRdRp* mutants, with GO enrichment analysis highlighting abiotic and oxygen response (Figure 5D), and with KEGG enrichment in metabolic pathways and stress response (Figure 5D).

## 3. Discussion

In eukaryotes such as fungi, plants, and nematodes, RdRps are central to RNA silencing pathways, with function spanning gene expression modulation, antiviral defense, and epigenetic modifications [49]. In viruses, RDR serves as a key enzyme for viral RNA, facilitating replication and transmission. Plant mitoviruses, belonging to the mitoviridae family, are characterized by a single open reading frame encoding an RdRp within host mitochondria. Their impact on the hosts varies, for example, while some mitoviruses enhanced drought tolerance in Chenopodium quinoa by altering the mitochondrial proteome [50], others like PsMV2 increase the virulence of wheat stripe rust, severely affecting wheat growth [51]. This dichotomy suggests that the effects of mitovirus infection are context-dependent, likely related to host health and infection levels.

The presence of mitoviral sequences in plant nuclei, either through indirect infection or horizontal transfer from mitochondria, has been well-documented [44]. *mRdRp* (AT2G07749), a member of the mitovirus family located in the *Arabidopsis* nucleus [47], has an as-yet-unclear function in plant development and stress response. In *Arabidopsis,* six nucleus-encoded RdRp proteins have been extensively studied, each with specialized and partially overlapping roles. RDR3/4/5 seem to represent ancestral redundancies, while RDR1/2/6 has undergone neofunctionalization, participating in RNA silencing and antiviral immunity [52,53]. Among them, RDR6 is particularly versatile, involved not only in antiviral defense but also in developmental regulation and stress response [54,55]. For instance, RDR6 is crucial for wheat anther development [51], and GhRDR6 has a pivotal role in cotton defense responses and development [56], and in rice, OsRDR6 is required for the formation of double-strand breaks during meiosis, essential for viable pollen production [52]. However, MtRDR6 impairs the production of endogenous phased siRNAs without affecting development [53]. Multiple alleles of RDR6 proteins have been identified across species, underscoring their importance in small RNA biogenesis and RNA-directed DNA methylation-mediated gene silencing.

In this study, we generated *mRdRp* mutants using the CRISPR-Cas9 or RNAi, uncovering a previously unreported role of *mRdRp* in embryo development. Our findings show that mRdRp is indispensable for embryo development, as loss-of-unction mutants exhibit high sterility and increased stress vulnerability; moreover, we found a significant elevation of small RNA levels in the mitochondria of *mRdRp* mutants. This is a novel and significant finding. Small RNAs can be classified into different types, such as microRNAs (miRNAs) and small interfering RNA (siRNAs). siRNAs can be derived from double-stranded RNA precursors and play roles in RNA-induced gene silencing pathways [57]. The origin of these mitochondrial small RNAs is likely multifaceted. Some may be encoded by mitochondrial genes themselves. For example, several mitochondrial coding genes, including ATMG00640, ATMG00650, ATMG01080, ATMG01360, and ATMG00480, show high transcriptional activity and generate substantial amounts of small RNAs (Figure 5A). In addition, certain non-coding regions of the mitochondrial genome also produce high levels of small RNAs. These observations could be attributed to the aberrant activation of two mitovirus-derived sequences (ATMG01410 and ATMG00490) integrated in the mitochondrial genome (Figure 5A). While the functions of these small RNAs remain elusive, their accumulation is likely to perturb normal mitochondrial physiology, contributing to the stress-sensitive and sterile phenotypes observed in *mRdRp* mutants. Thereby, mRdRp loss drives mt-sRNA accumulation, which in turn mediates the observed phenotypes through two interconnected mechanisms: (1) mt-sRNA-mediated autoregulation of mitochondrial function. Elevated mt-sRNA targeting respirator genes (ATP synthase and COXI) may suppress the translation or stability of their cognate transcripts, impairing mitochondrial respiration-consistent with the fragmented mitochondria and rotenone hypersensitivity of *mRdRp* mutants. (2) Crosstalk with nuclear RNA silencing. Upregulated miR1919-Y and downregulated AGO3 disrupt nuclear RISC activity, leading to the misregulation of stress-responsive genes and exacerbating sensitivity to ABA. Together, these findings establish mRdRp as a critical regulator of intercompartmental sRNA homeostasis, linking mitochondrial function to nuclear gene expression and plant phenotypic plasticity.

Regarding the *mRdRp* segments similar to mitoviruses sequences, they originate from ancient viral infections [58] or the horizontal transfer of mitochondrial DNA [59]. These capture sequences could confer evolutionary advantages or have been co-opted for developmental functions, which is in line with the theory of virus–host co-evolution [58]. Nemerous finding indicate that viruses are integral to the plant ecosystem [60,61], promoting host adaptation and often evolving from parasitism to mutualism [62]. Plants have developed a sophisticated system to maintain a balance of viruses’ presence with growth, and the RdRp plays a key role in coordinating this process.

## 4. Materials and Methods

### 4.1. Plant Material

Plant growth medium and conditions were as described previously [63]. *Arabidopsis* seeds were surface-sterilized with 70% ethanol for 1–2 min, then sown on 0.5× MS medium with 0.8% agar containing 2% sucrose. Plants were grown in a climate room under a 16 h light/8 h dark daily cycle at 20 ± 2 °C. For ABA or other chemical treatments, 3-day-old seedlings were transferred to a fresh 0.5× MS medium supplemented with the following regents at their respective final concentrations, 50 μM ABA, 10 μM malic acid, or 10 μM rotenone. The concentrations of these reagents were determined based on preliminary experiments, as described in our previous research. A solvent control group was also included, where seedlings were transferred to a fresh 0.5× MS medium containing the same volume of dimethyl sulfoxide (DMSO) as used in the reagent stock solutions. All treated and control seedlings were maintained in a climate room under the same 16 h light/8 h dark daily cycle at 20 ± 2 °C for a treatment duration of five days. After the five-day treatment, seedlings were photographed and subsequent experimental analyses were performed.

### 4.2. Vector Construction and Arabidopsis Transformation

cDNAs encoding *mRdRp* were amplified via PCR and cloned into the pCB302 plasmids backbone to generate two fusion constructs: *p35S:eGFP-mRdRp* or *p35S:mRdRp-mCherry*, respectively. For CRISPR/Cas9-mediated *mRdRp* knockout, synthetic guide RNA (sgRNA) sequence targeting the *mRdRp* coding region were designed and inserted into the pPTG vector as described previously [64]. For *mRdRp* knockdown, artificial microRNA (amiRNA) sequences specific to *mRdRp* were inserted into the HBT vector and then cloned into the pCB302 plasmid to generated *amiR-mRdRp*. Notably, In the *amiR-mRdRp* vector, the amiRNA cassette was inserted into the second exon of *GFP* gene. This GFP marker enables visual confirmation of vector transformation and expression in recipient plants. All recombinant plasmids were verified by Sanger sequencing and then transformed into Agrobacterium tumefaciens strains GV3101. Arabidopsis transformation was performed using the floral dip method.

### 4.3. Subcellular Localization Assays

Arabidopsis mesophyll protoplasts were isolated following Yoo [65] and Li [66] with slight modifications. The *p35S:eGFP-mRdRp* or *amiR-mRdRp* plasmids were transfected into protoplasts from wild-type plants. After transfection and incubation for 12 h in the dark at 22 ± 1 °C. Subcellar localization and mitochondrial morphology were visualized using a confocal laser scanning microscope (LSM800, Carl Zeiss, Oberkochen, Germany) with excitation/emission wavelengths of 488/509 nm for GFP and 587/610 nm for MitoTracker. For mitochondrial observation, protoplasts were stained with one μM MitoTracker Red CMXRos (Thermo Fisher Scientific, Waltham, MA, USA) for 3~5 min before imaging.

### 4.4. RNA Isolation and Transcriptome Analysis

Total RNA was extracted from five-day-old seedlings, as previously described, and measured on a NanoDrop 2000 (Thermo Fisher Scientific, Waltham, MA, USA) [63]. Subsequently, the RNA sequencing was conducted by GENE DENOVO (Guangzhou, China; https://www.genedenovo.com/). Raw data were processed and differentially expressed genes (DEGs) were identified using DESeq2 v1.34.0 with thresholds of |log_2_(fold change)| > 1 and adjusted *p*-value < 0.05. Functional annotation of DEGs was performed via Gene Ontology (GO) and Kyoto Encyclopedia of Genes and Genomes (KEGG) pathway analysis on the OmicShare platform (https://www.omicshare.com/index.php, accessed on 1 March 2025), with statistical significance defined as adjusted *p* < 0.05.

### 4.5. Statistical Analysis

The experimental data were collected and statistically analyzed using GraphPad prism8.0.1. Quantitative results are presented as mean ± SD of determination on at least three individual samples. Means were compared using the one-way ANOVA analysis of variance or multiple comparison tests. Significance was assigned at *p* < 0.01.

## 5. Conclusions

Our study significantly advances the understanding of mRdRp’s function in plant development. By demonstrating its essential role in embryo development and its connection to mitochondrial small RNA regulation, we have filled a knowledge gap in the field. The discovery of the role of mitochondrial small RNAs in *mRdRp* mutants provides a new perspective on plant-stress interactions. Future research could focus on developing molecular markers based on these small RNAs or *mRdRp* itself to screen for plants with enhanced stress tolerance. Additionally, further exploration of the relationship between mRdRp and other components of the RNA-silencing pathway may lead to the development of new genetic engineering strategies to improve crop performance. Overall, our findings not only contribute to fundamental plant biology knowledge but also hold promise for practical applications in agriculture.

## Data Availability

Data are contained within the article.
